# How do German bilingual schoolchildren process German prepositions? – A study on language-motor interactions

**DOI:** 10.1371/journal.pone.0193349

**Published:** 2018-03-14

**Authors:** Daniela Katharina Ahlberg, Heike Bischoff, Jessica Vanessa Strozyk, Doreen Bryant, Barbara Kaup

**Affiliations:** 1 LEAD Graduate School, Eberhard Karls University, Tübingen, Germany; 2 Institute of German Language and Literature, Eberhard Karls University, Tübingen, Germany; 3 Department of Psychology, Eberhard Karls University, Tübingen, Germany; Universite de Geneve, SWITZERLAND

## Abstract

While much support is found for embodied language processing in a first language (L1), evidence for embodiment in second language (L2) processing is rather sparse. In a recent study, we found support for L2 embodiment, but also an influence of L1 on L2 processing in adult learners. In the present study, we compared bilingual schoolchildren who speak German as one of their languages with monolingual German schoolchildren. We presented the German prepositions *auf* (*on*), *über* (*above*), and *unter* (*under*) in a Stroop-like task. Upward or downward responses were made depending on the font colour, resulting in compatible and incompatible trials. We found compatibility effects for all children, but in contrast to the adult sample, there were no processing differences between the children depending on the nature of their other language, suggesting that the processing of German prepositions of bilingual children is embodied in a similar way as in monolingual German children.

## Introduction

In the last decades, the idea that language learning and comprehension do not take place in an amodal, abstract, symbolic module separated from other modules in the brain, but are instead closely connected to perception and action has gained much attention (embodied cognition [1–2]). This view has been supported by a large number of empirical studies. For instance, neurophysiological studies have shown that the comprehension of action verbs activates areas of the motor cortex that are associated with the actual execution of the corresponding actions. For example, a word such as *kick* activates the leg-area of the motor cortex, whereas for a word such as *pick* the hand-area gets activated (somatotopic activation [3]; for similar findings see also [4–12]). Furthermore, on the behavioural level, studies have shown compatibility effects between language processing and action or perception. For example, reading words referring to entities with a typical location in the upper vs. lower part of the world (e.g., *sun* vs. *root*) leads to faster motor responses in a compatible direction (e.g., upward response to *sun*) than in an incompatible direction [13]. Similar compatibility effects between word meaning and response direction have also been shown for hand- vs. foot-associated words [14] and emotion words [15].

Such findings have often been interpreted within the framework of the theory of experiential traces by Zwaan and Madden [16]. This theory states that interactions with the world leave experiential traces in the brain, which are later reactivated when a person encounters linguistic stimuli referring to the respective objects, situations, and events. Thus, language comprehension can be understood as a reactivation of those experiential traces. Imagine the following situation as an example: A child focuses his or her attention on a certain object, for instance the neighbour’s dog, by looking or pointing at the dog. In many cases, the child will receive an explanation of the situation whereby the linguistic label of the object (i.e., the word *dog*) will be uttered. This situation constitutes a typical learning situation in native language (L1) acquisition. According to the experiential traces theory, such co-occurrences of linguistic labels and their referents are crucial for learning. Through frequent co-occurrence, experiential traces of linguistic labels get connected to the experiential traces of their referents such that later on the reactivation of the latter traces provide the basis for the comprehension of the linguistic labels [16]. Although our example focuses on nouns, similar assumptions can be made for other word types, as for instance spatial prepositions such as *on* or *under*. Consider a situation in which a child learns the word *under*. This will most likely be a situation in which someone refers to a relationship between a reference object and another object (e.g., *The cat is under the sofa*) or in the context of a request for putting a reference object into a particular spatial location (e.g., *Put the box under the table*). Experiencing situations with this kind of co-occurrence will over time lead to a rich set of experiential traces that presumably get reactivated whenever the spatial preposition is encountered, and thus, provide the basis for its comprehension.

The question arises why cognition should be represented in the same way as perception and action. From an evolutionary perspective, one possibility is that existing neural networks (i.e., sensorimotor networks) are used for relatively new functions such as language processing [17–18]. Another idea is that meaning cannot be stored in an exclusively amodal manner since such a system would be self-referential. That is to give meaning to one symbol it would refer to other symbols, which then would have to be explained through another set of symbols and so on. In order to give meaning to symbols, they have to be grounded in experience. Therefore, a different kind of representation format is needed. The latter view has predominated the language comprehension literature in recent years (for reviews see [2, 19–21]). According to a strong version of this view, reading or hearing words will automatically reactivate experiential traces, which are combined into a more complex experiential simulation during sentence comprehension. Furthermore, it is assumed that these simulations are the only possible representation of meaning and that, thus, comprehension is not possible without them. In other words, experiential simulations are functionally relevant for comprehension. The exact mechanisms behind comprehension are less clear than it seems though. For instance, it has recently been shown that the compatibility effects discussed above might actually not reflect sensory [22] or motor activation [23] after all. Furthermore, while there is evidence for compatibility effects on the sentence level [24–28], it is still unclear if these effects are due to simulation processes beyond the word level [29–30]. Additionally, the effects have been shown to strongly depend on the specific task and context [31–32]. For instance, it can be shown that the modality of a task facilitates modality-specific simulation (e.g., words like *red* would be more easily simulated in visual task requirements, whereas a word like *noisy* would profit from auditory task requirements) [31]. Last but not least, the functional relevance of experiential simulations, one of the core questions of this theory, is still under debate [33–34]. Thus, the possibility remains that experiential simulation is just a by-product of comprehension.

One prediction that follows from the functional relevance assumption is that language processing in a second language (L2) should also be based on experiential simulations. Evidence for this prediction is rather sparse. Nevertheless, a few studies using neural and behavioural paradigms suggest that L2 processing might also be embodied. For example, De Grauwe and colleagues [35] showed similar activation patterns in motor and sensorimotor regions for Dutch L1 and L2 speakers (with German L1) during a lexical decision task using motor verbs. Furthermore, Dudschig, de la Vega, and Kaup [36] reported compatibility effects between the implied location of a word’s referent and response direction for L2 speakers. In one of their experiments, German native speakers who learned English as L2, performed a Stroop-like task [37] in which they responded to English nouns, the referents of which are typically either found in an upper or in a lower location in the world (e.g., *star* and *root*, respectively). Depending on the font colour, participants responded either with an upward or a downward movement of the arm, resulting in compatible and incompatible trials. Their results revealed response facilitations for compatible (e.g., upward response to *star*) compared to incompatible trials (e.g., downward response to *star*), just as had been found for L1 speakers [13], indicating that location information is not only automatically activated in L1 processing, but in L2 processing as well. This finding suggests that the reactivation of experiential traces is not restricted to L1 processing. However, at this point it remains unclear whether the experiential traces that already exist for the L1 are just extended to the L2, or whether a completely different system of experiential traces is used for the L2.

In the examples described above, the categorizations in the target and the source language were similar, that is each word in the L2 had a more or less direct counterpart in the L1. In this case, it is possible to integrate the newly learned words into the already existing network of experiential traces. Inevitably, this leads to the question what happens when the words of an L2 cannot be directly linked to an earlier developed experiential trace. One prominent example for this is the categorization of space by means of spatial terms. Research on the use of spatial terms in different languages not only shows that languages use spatial terms in different ways grammatically (some use them as prepositions, e.g., English, Russian, and German, others use them as postpositions, e.g. Turkish and Korean), but also that categorizations of space are very different across languages [38–39]. For instance, Turkish and German use the same system of co-ordinate axes to structure space (e.g., upper/lower, front/back, and left/right subspaces). But while German makes a further distinction with regard to the upper subspace, Turkish does not [40]. The division of the upper subspace in German depends on the feature ‘contact’: If an object is situated above another object and contacts it, this is described by using *auf* (*on*); in contrast, if the described object has no contact to the other object the preposition *über* (*above*, *over*) is used. In Turkish there is no such division of the upper subspace, and accordingly, the feature ‘contact’ becomes irrelevant for the description of these spatial configurations. Rather, the words *üstünde* or *üzerinde*, which can be used interchangeably, are used in both sorts of configurations [40]. A similar difference can be found in many other languages as well. For example, Russian and English differentiate the upper subspace in a similar way as German does, while Korean and Japanese, similar to Turkish, do not make the distinction [40–41]. This difference in the categorization of space between the languages might be responsible for certain learning difficulties that are associated with the learning of spatial categories [42–44] and German spatial prepositions in particular [45–47].

To shed more light on this issue, we previously conducted a behavioural study to investigate how speakers of different native languages process the German prepositions *auf*, *über*, and *unter* [48]. We tested adult participants, who had acquired German as L1 and compared them to adults who acquired Russian or English as L1 and German as L2, as well as to adults who acquired Turkish or Korean as L1 and German as L2. We focused on these languages, because of their categorization differences, as described above. Similarly to Dudschig and colleagues [36], we presented participants with a Stroop-like task in which they responded to words presented on the screen either with an upward or a downward arm movement depending on the font colour of the presented words. Instead of nouns referring to entities typically found in the upper or lower part of the world, we used the prepositions *auf*, *über*, and *unter* mentioned above. This setup resulted in compatible trials (e.g., *über* followed by an upward movement) and incompatible trials (e.g., *über* followed by a downward movement). We found a significant compatibility effect, with faster response times for compatible compared to incompatible trials, for all participants regardless of their native language. This finding supports the above-mentioned hypothesis that L2 processing is embodied and that comprehension relies on the reactivation of experiential traces. In addition, we found differences in the processing of the words *auf* and *über* depending on the native language of our participants: While the German L1 speakers, as well as the speakers with Russian or English as L1 showed a compatibility effect for *über* (faster responses when responding with an upward than with a downward movement) the Turkish and Korean L1 speakers did not. They did however show a compatibility effect for *auf* just as the Russian and English L1 speakers. Unexpectedly, the native speakers of German showed no compatibility effect for *auf*. For a detailed discussion of this finding see our previous study [48]. These differences presumably reflect the fact that Turkish and Korean (in contrast to German, Russian, and English) do not differentiate between +/- contact in the vertical dimension, and thus do not distinguish between the meaning of *auf* and *über*. As a result, these speakers probably transfer all their experiential traces to the preposition *auf* which is much more frequent than *über* [49] and is also acquired earlier during language learning [45]. This explains why they show experience-based spatial compatibility effects for *auf* but not for *über*.

These findings are in line with Slobin’s thinking-for-speaking hypothesis [50], according to which once a category is set in the L1, it shows a strong resistance to posterior restructuring. The way our L1 is categorized influences our perception and guides our attention in the L2. The perception of the categorization in the L2 is thereby directly influenced by the L1 [51]. Taking this view and our previous results together, it seems likely that when learning a new language, the words of the L2 will be connected with the experiential traces of the L1, in a first step. Then, in a second step, new experiential traces have to be developed for configurations in the L2 that are not present in the L1.

The adults tested in our previous study [48] mainly learned their L2 after the age of twelve. Therefore, the semantic categorizations of the L1, as well as the connected experiential traces were probably already very strongly consolidated. As is well known, the age at which a language is being acquired plays an important role in the acquisition process [52–54]. Therefore, in the current study we aimed at investigating compatibility effects in bilingual participants who learned German as well as at least one other language (OL) before the age of six. We were interested in whether these bilinguals process German spatial prepositions in a similar way as German monolinguals do, or whether their processing is influenced by the nature of the OL.

We conducted a Stroop-like task, similar to the task we used in our adult study, again concentrating on the prepositions *auf*, *über*, and *unter*. We compared three groups of children attending secondary school in Germany: the first group had only acquired German until the age of six. The second group had acquired German as well as at least one other language until the age of six, whereby the other language/languages were similar to German with respect to the division of the upper subspace. The third group had acquired German as well as at least one other language until the age of six, whereby the other language/languages were dissimilar to German with respect to the division of the upper subspace. Just as in the experiments with adult participants, we expect to find compatibility effects (i.e., faster responses for compatible compared to incompatible trials) in all groups. Children in secondary school should have already developed a network of experiential traces for spatial categories, as well as sufficient reading fluency [55], to show similar compatibility effects as adults. With regard to the processing of the individual prepositions, different scenarios are imaginable. First, semantic categorizations in the OL might be predominant despite the fact that German was acquired at an early age and participants have had several years of language contact with German. If so, it can be expected that the spatial system of the other language has an impact on the processing of spatial terms in German. In this case we expect different compatibility effects for different groups of speakers depending on the nature of their OL, just as in our study with adult participants. Second, early age of acquisition and several years of contact with German may have allowed the children to develop an independent spatial system for German even if it deviates from the spatial system in their OL. If so, we expect to find comparable compatibility effects for all groups of children, independent of the nature of their OL.

A further point we wanted to investigate is the role of language proficiency. Some authors hypothesize that language proficiency plays a major role in the development of experiential traces [35, 56]. Furthermore, in our study with adult participants [48], we found tentative evidence that experiential traces change over time with increasing proficiency. For instance, while for highly proficient participants a compatibility effect was found for *auf* as well as for *über*, in the mid-to-low proficient participants the compatibility effect was only found for *auf* but not for *über*. Thus, the pattern of results for highly proficient participants resembled the pattern of results of native speakers more closely than that of the mid-to-low proficient participants. This finding is in line with the findings of Bryant [45], according to which *auf* is learned earlier than *über*. However, the sample size in our previous study was not large enough to draw stable inferences after the sub-categorization into two proficiency groups. Therefore, we added an objective measure to assess language proficiency in the present study to further investigate this question.

## Method

### Participants

Four hundred and two schoolchildren of different secondary schools in Southern Germany took part in our experiment. Each class received a financial reward for their participation. The experimental testing was in agreement with the guidelines for good scientific practice at the LEAD Graduate School at the University of Tübingen, Germany. The study was approved by the ethics committee at the Faculty of Economics and Social Sciences, University of Tübingen. Prior to experiment participation the parents of our participants gave their written informed consent. Throughout the data acquisition the data were connected only by a participant code and at no point could the recorded data be associated with a participant’s name.

We grouped our participants into three groups: children who only acquired German as a native language and no additional language until the age of 6 (in the following: “German monolinguals”); children who learned German and at least one other language before the age of six, whereby the other language or the other languages split up the upper subspace with two different expressions just like German (e.g., Russian, English, Italian; in the following: “German bilinguals: similar OL”); children whose other language does not further distinguish the upper subspace (e.g., Turkish, Urdu, Swahili; in the following: “German bilinguals: dissimilar OL”). For an exact overview of the language group assignment see the S1 Table in the supplementary material. We had to exclude 19 participants from our sample, because we were not able to categorize them into one of these groups. Either we were not fully able to tell whether their OL is similar to or dissimilar from German due to the lack of information from the participants about their spoken dialects or their OLs conflicted with each other with respect to the categorization (e.g., English and Turkish; Turkish and Kurdish). In addition, we excluded 53 participants who committed errors on more than 20% of the trials, and 10 participants who responded faster than 100 ms in more than 20% of the trials. Although clearly instructed to use only their dominant hand to respond, some children could not be prevented from using both hands in the experiment (i.e. they used one hand to release the middle button and pressed the upper or lower button with the other hand almost instantaneously). This resulted in response times lower than 100 ms. To be sure to include only children who followed the instructions, we used this as an exclusion criterion.

The remaining 320 participants (*M*_*age*_ = 13.0 years, *SD*_*age*_ = 1.5 years, 166 male, 289 right-handed) were distributed over our three groups as follows: 130 German monolinguals (*M*_*age*_ = 13.0 years, *SD*_*age*_ = 1.5 years), 138 German bilinguals with a similar OL (*M*_*age*_ = 13.0 years, *SD*_*age*_ = 1.5 years), and 52 German bilinguals with a dissimilar OL (*M*_*age*_ = 13.1 years, *SD*_*age*_ = 1.7 years). For a more detailed overview on the distribution over class levels and school types see Table 1. All participants had normal or corrected-to-normal vision.

**Table 1:** Descriptive information about class level, school type, and bilingualism [57] of our participants.

Descriptives	Language Group		
	German Monolinguals	German Bilinguals: Similar-OL	German Bilinguals: Dissimilar-OL
Class Level (in %)			
5	10.8	18.1	19.2
6	25.4	23.9	19.2
7	16.9	17.4	11.5
8	26.2	22.5	28.8
9	20.8	18.1	21.2
School Type (in %)			
Werkrealschule	56.2	76.8	73.1
Gemeinschaftsschule	29.2	21.0	21.2
Realschule	14.6	2.2	5.8
Grade of Bilingualism (in %)			
Simultaneous Bilingualism		63.0	75.0
Early-Successive Bilingualism		11.6	11.5
Childlike L2 Acquisition		12.3	7.7
Not Specifiable		13.0	5.8

The categorization of bilingualism was made depending on the age of acquisition of German: 0–3 years–simultaneous bilingualism; 3–5 years–early-successive bilingualism; > 5 years–childlike L2 acquisition. A few children could not be classified, as their questionnaires were incomplete. In Germany, different types of secondary schools exist. Werkrealschule and Realschule offer secondary education for years 1–10, with a stronger focus on practical skills in the Werkrealschule. Additionally, the Gymnasium qualifies for University education. Gemeinschaftsschule is a school that serves as a combination of these three school types. Percentages within each group add up to 100%; deviations are due to rounding.

### Material

#### Language tests

To gain objective information about the children’s language proficiency of German, we conducted a “C-Test” [58–59]. This paper-pencil test measures general language skills based on four short texts with 20 gaps each (with a length of about half a word) that need to be completed. The scoring gives two measures: The word-recognition score and the accuracy score. The word-recognition score represents the number of correctly recognized words, regardless of their spelling accuracy. The accuracy score additionally takes the spelling into account. Both scores can reach a maximum of 80, one for each gap, while the accuracy score is typically lower than the word-recognition score. We used different tests for each class level to prevent floor or ceiling effects. The test for the fifth grade was taken from Baur, Chlosta, and Goggin [60]. The remaining tests for the 6^th^ to 9^th^ grade were provided by the same authors [61–64].

**Fig 1:**
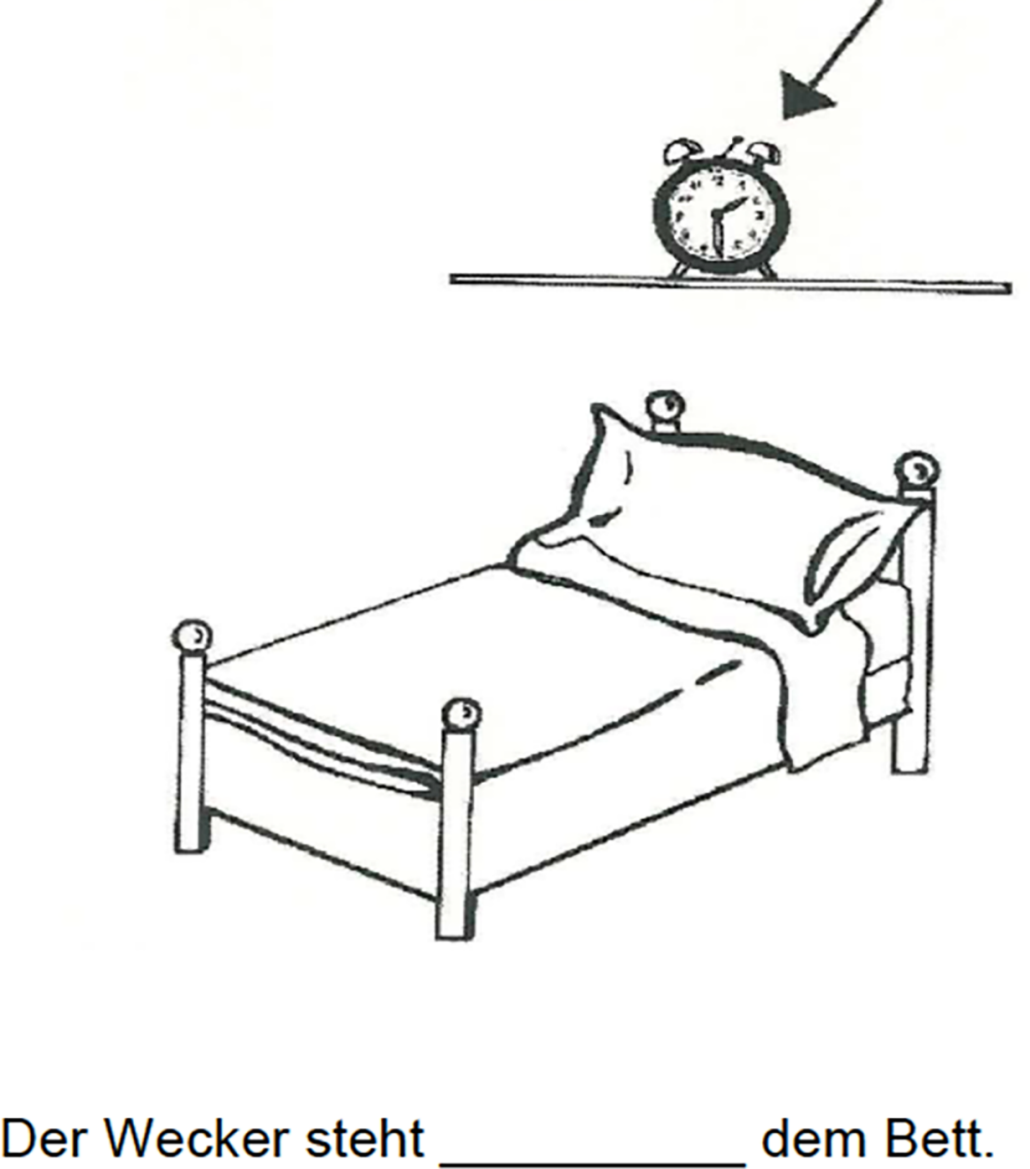
Example item from the used adaptation of the TRPS [45]. The child is expected to fill in the preposition *über* (*Der Wecker steht über dem Bett / The alarm clock is standing above the bed)*.

To get information about whether the children have acquired the meaning of the German prepositions used in the experiment, we used a paper-pencil adaptation of the Topological-Relations-Picture Series (TRPS) [65] on the basis of the adaptation of Bryant [45], who used a shortened version of this test and added different pictures to adapt it to typical German spatial configurations (see Fig 1 for an example item). We further shortened this set to 15 test pictures and one example item that was discussed with the children first. The gaps in the sentences needed to be filled with the prepositions *auf*, *über*, *unter*, *an*, and *in*, each three times. This resulted in a total maximum score of 15 and a maximum score of 9 for the prepositions *auf*, *über*, and *unter*. For the results of both tests see Table 2.

**Table 2:** Average scores on language tests, self-reported language contact/use, and socio-economic status of the language groups.

Tests	Language Group						
	German Mono-linguals	N	Bilinguals with similar OL	N	Bilinguals with dis-similar OL	N	
C-Test							
Word recognition Score (%)	89.2	130	80.0	136	76.7	52	
	(9.7)		(17.0)		(12.7)		
Accuracy Score (%)	79.5	130	67.1	136	62.8	52	
	(13.0)		(19.4)		(16.4)		
Prepositional Use							
*auf/über/unter–*Score (max. 9)	8.4	129	7.8	138	7.5	52	
	(1.0)		(1.7)		(1.7)		
Total Score (max. 15)	14.0	129	12.2	138	11.5	52	
	(1.4)		(2.6)		(2.7)		
Language Contact							
German Contact (1–5)			3.2	126	2.9	51	
			(1.3)		(1.1)		
OL Contact (1–5)			3.4	126	3.7	51	
			(1.2)		(1.2)		
Activities in OL (1–6)			1.5	124	2.2	49	
			(1.4)		(1.7)		
HISEI (16–90)	49.1	122	36.9	119	34.7	51
	(17.8)		(16.0)		(17.3)	

Standard deviations appear in parentheses below means. For specific information regarding the acquisition of these variables see the method section. Not all questionnaires and tests were fully filled in by all participants. Therefore, we provide information about the sample size in an extra column (N). HISEI = Highest International Socio-Economic Index of Occupational Status. OL = Other Language.

#### Questionnaires

We designed two questionnaires, one for the children and one for their parents. In these questionnaires we assessed the language background of the children, the origin of their parents and grandparents (country of origin and native language of parents and grandparents, as well as which languages the child speaks and how proficient he or she is in these languages), as well as the socio-economic status of the families in form of the HISEI (Highest International Socio-Economic Index of Occupational Status) [66]. Both questionnaires contained similar questions, but we adapted the wording and illustration to match the different target groups. By using questionnaires for children and their parents we wanted to increase the likelihood of obtaining the relevant information from at least one of the groups. For the categorization and the analyses we used the data given by the parents if available, otherwise we used the data given by the children. For the assessment of the children’s language contact, we included two questions. First, the children needed to indicate which languages they spoke with which persons. They were presented with a list of five persons or groups of persons (father, mother, siblings, other relatives, and friends). For each entry the children indicated which language they spoke to the respective person(s). Multiple answers were possible. We then counted how many times the children mentioned German for our German-contact measure and how many times the other language was mentioned for our other-language-contact variable. In the second question, we asked which languages they use in their free-time activities. Here the children were presented with six categories (watching TV, reading newspapers, reading books, reading on the Internet, listening to the radio, listening to music) and again they indicated which language or languages they used for these activities. We counted the number of other language indications only, as due to the living environment of the children, German was used in nearly all free-time activities. A summary of the results of these questionnaires can be found in Table 2.

#### Stimuli and apparatus

**Fig 2:**
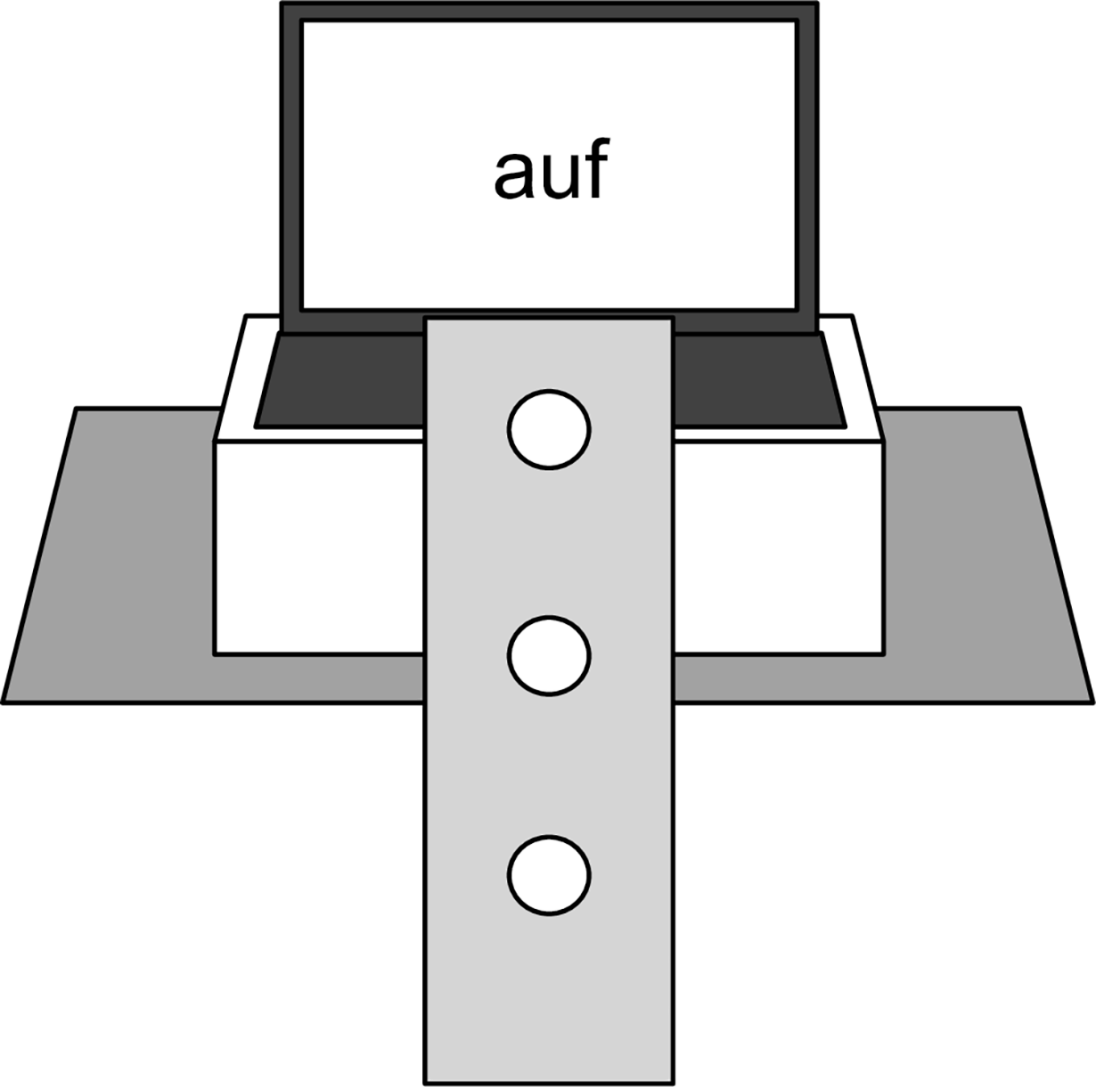
Experimental setup. The keyboard was implemented under a vertical plane in front of the participants. At the beginning of each trial, the participant pressed the middle key with their dominant hand. A response was made by releasing the middle button, pressing the upper or lower button, and returning back to the middle button.

Similar to our previous study [48], we concentrated on three German words serving as stimuli, namely *über* (*above*), *auf* (*on*), and *unter* (*below*). *Auf* and *über* served as referents for the upper dimension, which differ with respect to the feature +/- contact, and *unter* served as a referent for the lower dimension. As in our earlier study [48], we included the word *ab* (*down/off*) as a counterpart to *auf*, to balance the number of stimuli for the two different dimensions. The particle *ab* is part of the directional adverb *abwärts* (*downwards*). However, as the word *ab* is neither a spatial preposition nor is it used in explicit spatial configurations, we did not include it in our analyses but rather treated it as a filler item. Its spatial use is mostly restricted to its combination with *auf* (*auf und ab*–*up and down*). The four words were presented in four different font colours that have been proven to be easily distinguishable by most participants in a number of experiments in our lab (e.g., [13, 15]): blue (RedGreenBlue values (RGB) 0, 0, 255), orange (RGB 255, 128, 0), lilac (RGB 150, 0, 255), and brown (RGB 140, 80, 20). Each word appeared equally often in each colour. Responses were recorded using a PS/2 computer-keyboard adapted with a locally constructed overlay. For the experiment conduction we used LENOVO ThinkPad L530 notebooks. To enable the participants to view the screen, despite the height of the vertically mounted keyboard, we positioned the laptops on boxes on the tables right behind the keyboards. For the exact setup see Fig 2. The experiment was programmed with E-Prime® (Psychology Software Tools Inc., http://www.pstnet.com/E-Prime/e-prime.htm).

### Procedure and design

In the first school, where we tested 93 children in total, data were collected in two sessions on two separate days. However, since we lost data (e.g., the C-Test data of two participants) due to dropouts, all other data collections were done in one session. We tested each child in a session of maximally 45 minutes, 8 children in parallel. We conducted the experiment and the prepositional knowledge test right after each other in about 20 minutes of the session, while the language proficiency test took place in the remaining time. We balanced the order of those two parts; every session 4 children started with the experiment and 4 children did the language proficiency task. After half of the session they switched.

In the experiment proper, each trial started with a fixation cross, displayed in the centre position of the screen for 1000 ms. Afterwards the stimulus was presented in centre position, until the participant released the middle button or for a maximum of 2000 ms. Right after the button release, a blank screen was shown until the second response, a button press of the upper or lower button, or for a maximum of 3000 ms. Between trials, a white screen was shown for 1000 ms. Note that in the first data acquisition sessions, we showed the stimulus and the blank screen until response execution without a predetermined cut-off time. Since some children exceeded our maximum testing time of 45 minutes (determined by the length of one school lesson) in this setup, we decided to include an automatic cut-off to improve the motivation of the children. For the first 93 participants, we recoded reaction times exceeding the cut-off as errors.

The participants used a response box with three buttons for their task as can be seen in Fig 2. They were asked to only use their dominant hand (i.e., left hand for left-handers; right hand for right-handers) throughout the whole experiment. At the beginning of each trial, they were asked to push down the middle button and to keep it pressed until the stimulus appeared on the screen. When they had decided whether to press the upper or lower button depending on the font colour of the presented word, the participants were to release the middle button and press the upper or lower button instead, before returning to the middle button. The participants were instructed to respond to the font colour of the stimuli as quickly and accurately as possible.

The upper and the lower button were each associated with two of the four possible colours. This mapping of colours to response direction was balanced across participants. All possible colour pairs occurred equally often and were randomly assigned to the two buttons.

Each word was presented 40 times, resulting in a total number of 160 trials, which were subdivided into 2 experimental blocks. The experiment started with a practice block, consisting of 60 trials, in which we presented stimuli different from the experimental stimuli in the four colours. In all the blocks the participants received visual feedback about response accuracy (the German word for correct or incorrect presented at the centre of the screen) after each trial in order to keep them motivated and as a reminder of the correct response direction.

The design was a 3 (stimulus: *auf*, *über*, *unter*) x 2 (response direction: upward vs. downward) x 3 (language group: German monolinguals vs. German bilinguals with similar OL vs. German bilinguals with dissimilar OL) design with stimulus and response direction as within-subjects factors and language group as between-subjects factor. The dependent variables were the release time of the middle button, as well as the errors.

## Results

The mean error rate was 6.8%. For the analysis of reaction times, errors and trials with release responses or movement responses faster than 100 ms were excluded. Responses deviating by more than 3 *SD*s from the mean for each participant and condition (stimulus x response) were also excluded. This outlier elimination reduced the data by 2.6%. In the analysis of errors no data were excluded from the analyses.

### General analysis

#### Reaction times

**Fig 3:**
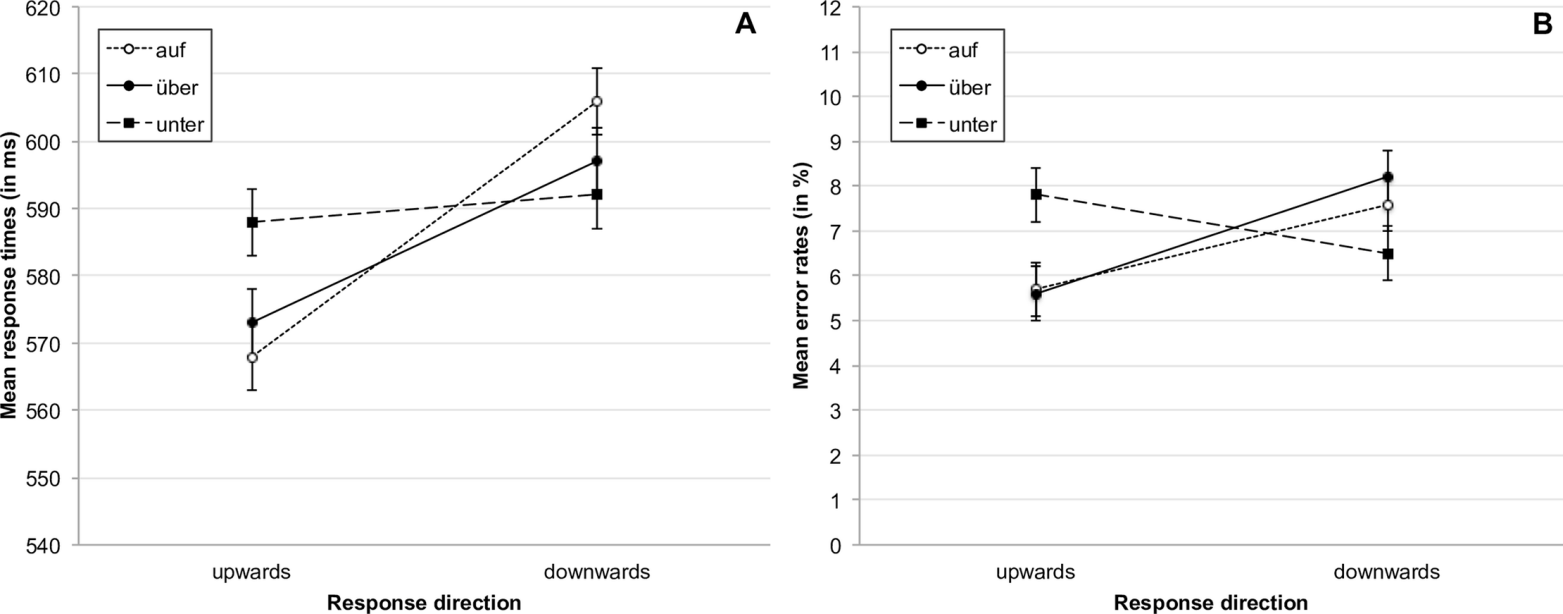
**Mean response times for correct responses (A) and mean percentage of errors (B) as a function of response direction and stimulus for all participants.** Error bars represent 95% confidence intervals [67].

In our analysis, we obtained a significant interaction effect between stimulus and response direction, *F*(2, 634) = 18.54, *p* < .001, η_p_^2^ = .055, reflecting the expected compatibility effect. As can be seen in Fig 3A, responses in compatible trials (e.g., upward response to *auf*) were overall faster than responses in incompatible trials (e.g., downward response to *auf*). In addition, we found a significant main effect of response direction, *F*(1, 317) = 49.42, *p* < .001, η_p_^2^ = .135, with overall faster upward than downward responses. When looking at the individual stimuli separately, we found significant differences between compatible and incompatible responses for the prepositions *auf*, *t*(319) = -8.98, *p* < .001, and *über*, *t*(319) = -5.37, *p* < .001, but not for *unter*, *t*(319) = -1.49, *p* = .136.

The interaction between stimulus, response direction, and language group was not significant, *F*(4, 634) = 1.31, *p* = .264, η_p_^2^ = .008, indicating that all language groups showed similar compatibility effects. The main effects of stimulus, *F*(2, 634) = 1.03, *p* = .358, η_p_^2^ = .003, and language group, *F*(2, 317) = 1.01, *p* = .364, η_p_^2^ = .006, as well as all other interactions involving the between subjects factor language group revealed no significant effects (stimulus x language group, *F*(4, 634) = 1.52, *p* = .196, η_p_^2^ = .009; response direction x language group, *F*(2, 317) = 1.33, *p* = .267, η_p_^2^ = .008).

#### Error rates

The error analysis overall supported the results of the analysis of the response times: We found a significant interaction between Stimulus and Response Direction, *F*(2, 634) = 16.66, *p* < .001, η_p_^2^ = .050, indicating that more errors were made in incompatible trials compared to compatible trials (see Fig 3B). The main effect of response direction, *F*(1, 317) = 9.73, *p* = .002, η_p_^2^ = .030, was also significant: The participants made more errors on downwards trials (7.4%) than on upwards trials (6.4%). In the separate analyses for the individual prepositions, we found significant differences between compatible and incompatible responses for the preposition *auf*, *t*(319) = -3.54, *p* < .001, and *über*, *t*(319) = -4.56, *p* < .001, as well as for *unter*, *t*(319) = 2.48, *p* = .014.

Just as in the reaction times analysis, the compatibility effect did not differ depending on the language group: The interaction between stimulus, response direction, and language group was not significant, *F*(4, 634) = 1.24, *p* = .291, η_p_^2^ = .008. The interaction between response direction and language group, *F*(2, 317) = 1.16, *p* = .314, η_p_^2^ = .007, the interaction between stimulus and language group, *F*(4, 634) = 1.41, *p* = .230, η_p_^2^ = .009, and the main effects for language group, *F* < 1, and stimulus, *F*(2, 634) = 1.00, *p* = .368, η_p_^2^ = .003, were also not significant.

Taken together, the fact that we found compatibility effects in the reaction times and the error rates independent of language group implies that experiential traces got reactivated not only in the group of German monolinguals, but also in the groups of German bilinguals with similar and dissimilar OL. This is exactly what we expected. However, as we found differences between the language groups for adults [48], we nevertheless conducted separate analyses for all three groups. This seemed reasonable since the main objective of our study was to investigate differences and similarities between the language groups and we wanted to make sure that we did not overlook more subtle differences.

### Separate analyses for the different language groups

#### Reaction times

In all groups we found the same pattern of results as in the main analysis. We found a significant interaction between stimulus and response direction (German monolinguals: *F*(2, 258) = 4.46, *p* = .012, η_p_^2^ = .033; similar-OL: *F*(2, 274) = 10.13, *p* < .001, η_p_^2^ = .069; dissimilar-OL: *F*(2, 102) = 6.43, *p* = .002, η_p_^2^ = .112) and a significant main effect of response direction (German monolinguals: *F*(1, 129) = 12.49, *p* < .001, η_p_^2^ = .088; similar-OL: *F*(1, 137) = 39.66, *p* < .001, η_p_^2^ = .225; dissimilar-OL: *F*(1, 51) = 10.12, *p* = .002, η_p_^2^ = .166), while the main effect of stimulus was not significant (German monolinguals: *F* < 1; similar-OL: *F*(2, 274) = 2.31, *p* = .101, η_p_^2^ = .017; dissimilar-OL: *F* < 1).

**Fig 4:**
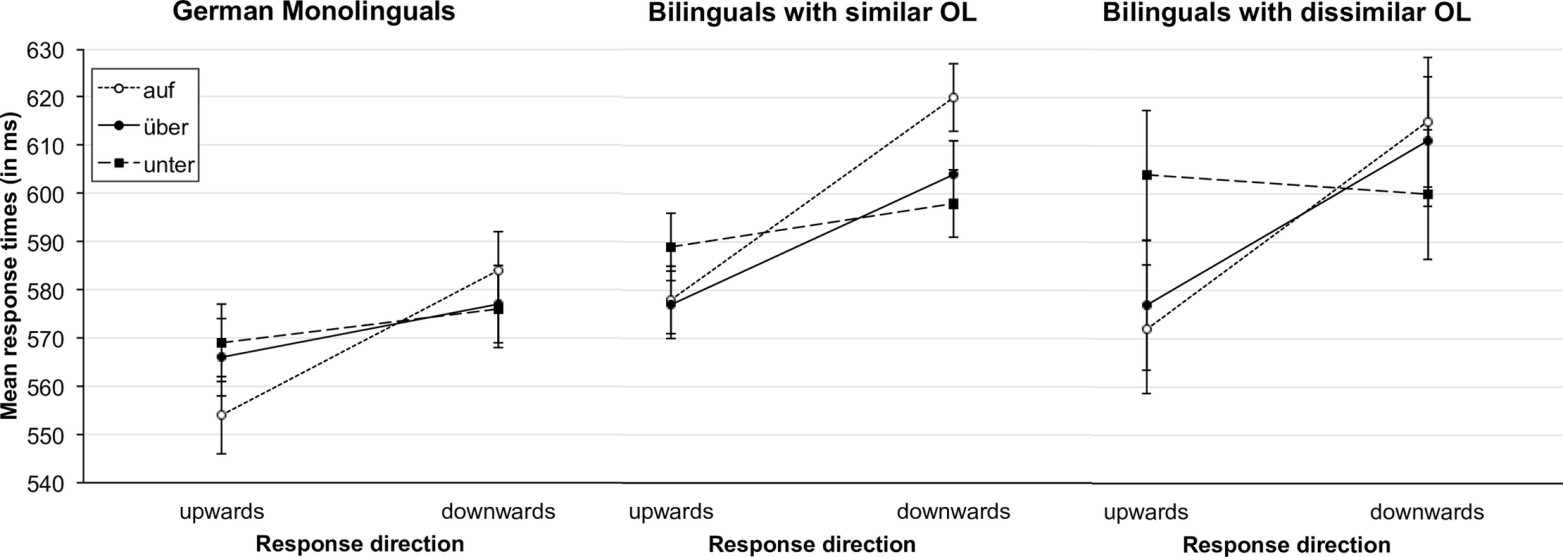
Mean response times for correct responses as a function of response direction and stimulus for the different language groups separately. Error bars represent 95% confidence intervals [67].

Although all groups showed a similar pattern of results, Fig 4 also reveals some small differences with regard to the compatibility effects of the individual prepositions. Therefore, we compared compatible and incompatible response times for the different stimuli for each language group separately. The results can be found in Table 3. In all three groups there was a significant compatibility effect for *auf* but not for *unter*. The only difference between the groups was that the compatibility effect for *über* was significant for both the similar and the dissimilar-OL group, while it was only marginally significant for the German monolinguals.

**Table 3:** Individual comparisons of the RTs for compatible and incompatible trials per language group.

	*df*	*t*	*p*
German monolinguals			
*auf*	129	-4.43	< .001
*über*	129	-1.90	.060
*unter*	129	-1.00	.321
similar-OL			
*auf*	137	-6.81	< .001
*über*	137	-4.49	< .001
*unter*	137	-1.67	.097
dissimilar-OL			
*auf*	51	-4.39	< .001
*über*	51	-2.88	.006
*unter*	51	0.32	.748

#### Error rates

**Fig 5:**
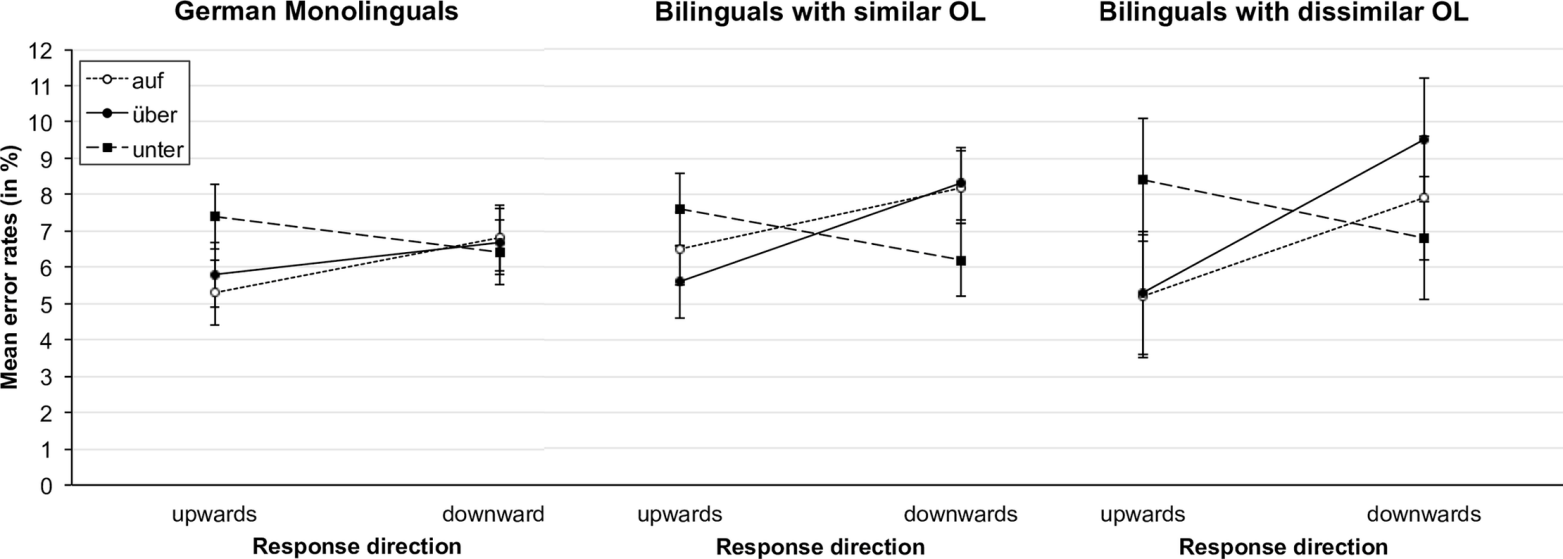
Mean percentage of errors as a function of response direction and stimulus for the different language groups separately. Error bars represent 95% confidence intervals [67].

The analysis of the errors overall supported the findings of the reaction times analysis, as the interaction between stimulus and response direction was significant in all groups (German monolinguals: *F*(2, 258) = 3.70, *p* = .026, η_p_^2^ = .028; similar-OL: *F*(2, 274) = 8.21, *p* < .001, η_p_^2^ = .057; dissimilar-OL: *F*(2, 102) = 5.58, *p* = .005, η_p_^2^ = .099), whereas the main effect of stimulus was not significant (German monolinguals: *F*(2, 258) = 1.93, *p* = .147, η_p_^2^ = .015; similar-OL: *F* < 1; dissimilar-OL: *F* < 1). The main effect of response direction was significant for both the similar- and the dissimilar-OL group (similar-OL: *F*(1, 137) = 4.11, *p* = .044, η_p_^2^ = .029; dissimilar- OL: *F*(1, 51) = 4.85, *p* = .032, η_p_^2^ = .087) but not for the German monolinguals (*F* < 1). The mean error rates for the different language groups are displayed in Fig 5.

The results of the separate analyses of the compatibility effects for the different stimuli and language groups can be found in Table 4. For the German monolingual speakers significant differences were obtained only for *auf*, but not for *über*, and *unter*. For the other two groups, the differences between compatible and incompatible responses were significant for *über*, but not for *unter*. Additionally, significant differences were obtained for the word *auf* for the similar-OL group, while the difference was only marginally significant for the dissimilar-OL group.

**Table 4:** Individual comparisons of the error rates for compatible and incompatible trials per language group.

	*df*	*t*	*p*
German monolinguals			
*auf*	129	-2.12	.036
*über*	129	-1.17	.244
*unter*	129	1.40	.166
similar OL			
*auf*	137	-2.10	.038
*über*	137	-3.81	< .001
*unter*	137	1.64	.104
dissimilar OL			
*auf*	51	-1.99	.052
*über*	51	-3.12	.003
*unter*	51	1.25	.218

### Analysis of language proficiency

**Fig 6:**
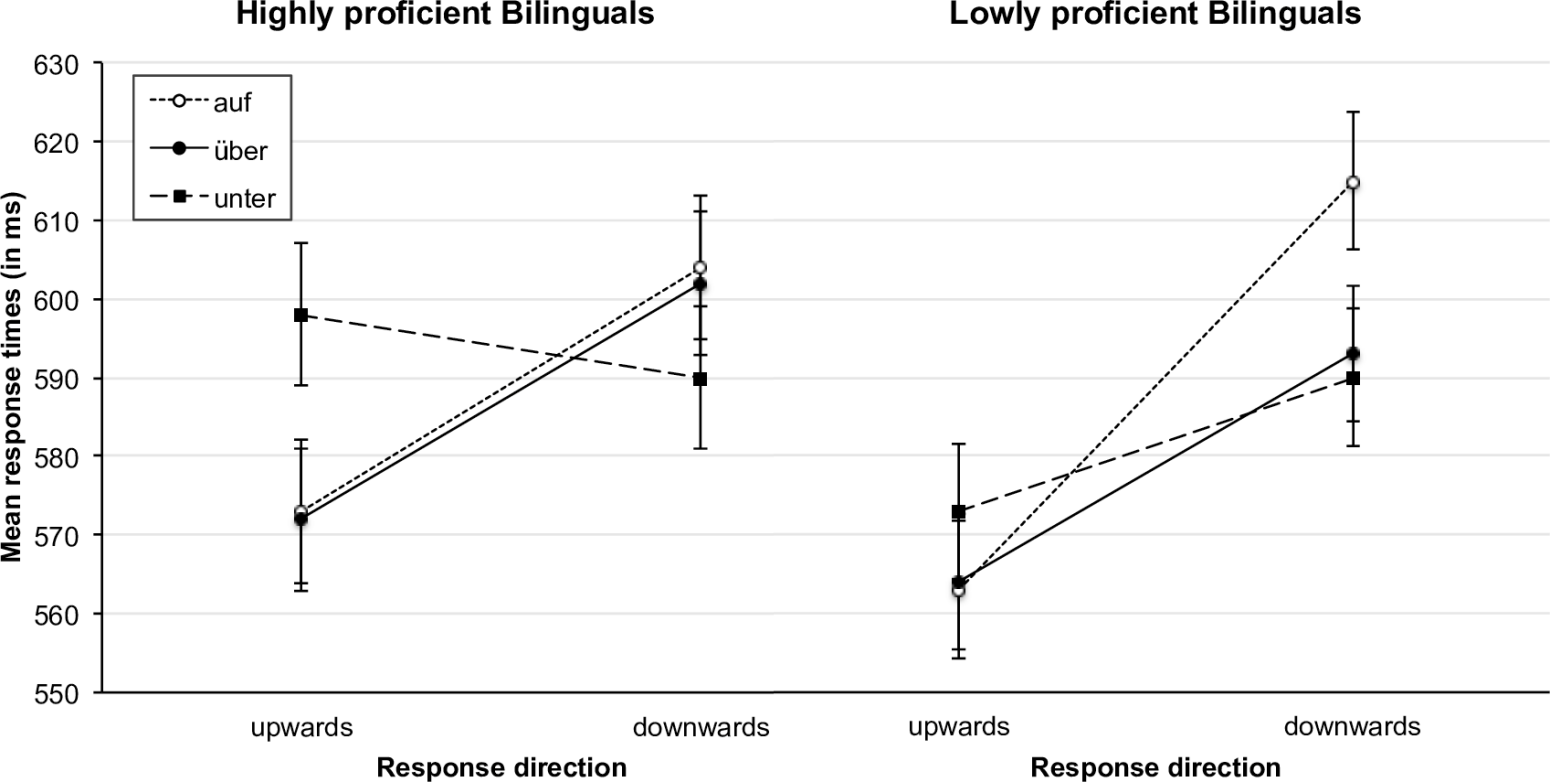
Mean response times of the high-proficiency and the low-proficiency group for correct responses as a function of response direction and stimulus. Error bars represent 95% confidence intervals [67].

**Fig 7:**
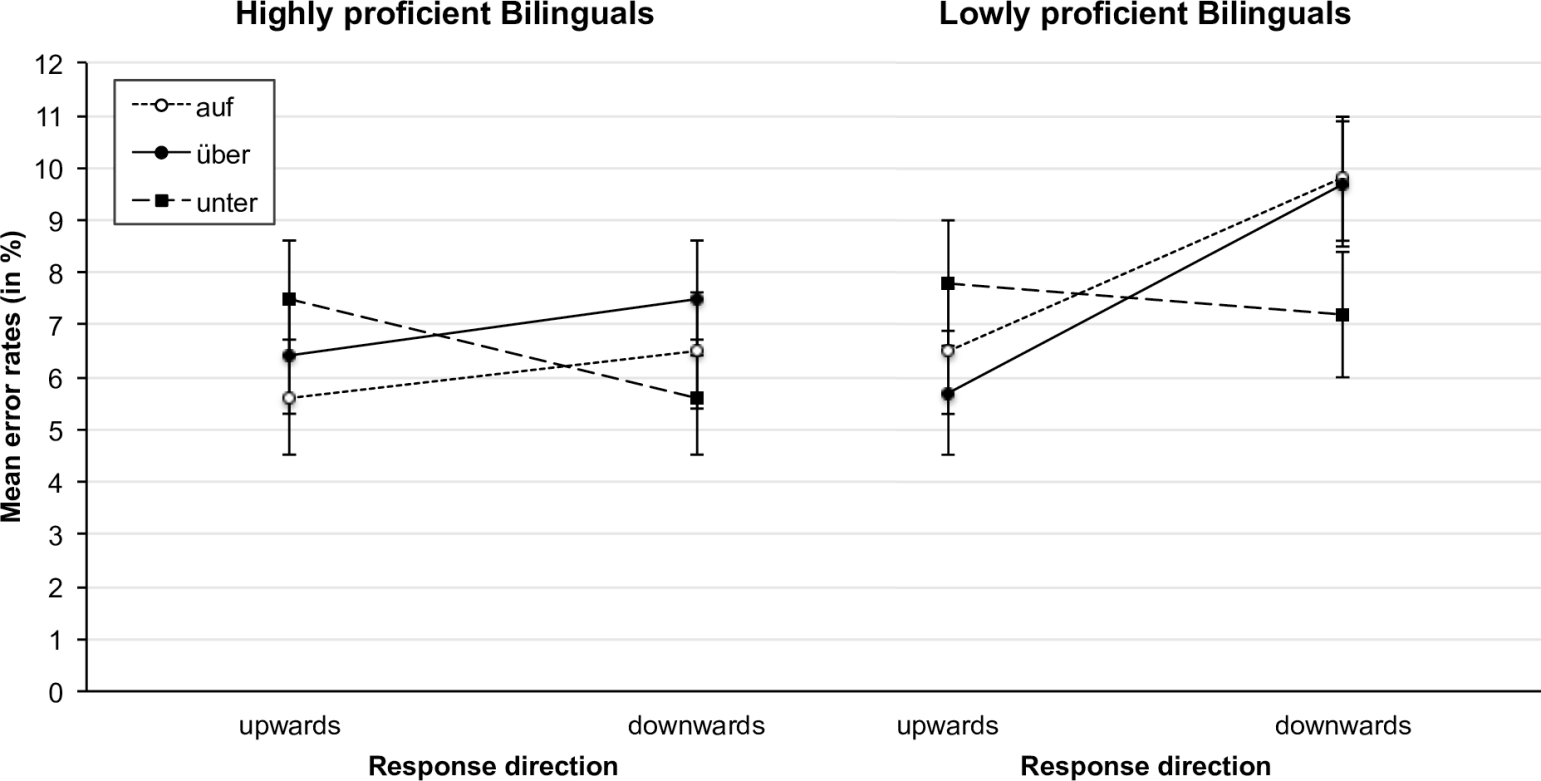
Mean percentage of errors of the high-proficiency and the low-proficiency group as a function of response direction and stimulus. Error bars represent 95% confidence intervals [67].

As mentioned above, a second objective of the current study was to investigate the role of language proficiency in bilingual language processing of prepositions. For that reason, we pooled both bilingual groups together and conducted a median-split based on the word-recognition percentage score of the C-Test (N = 190; two C-Tests were missing due to participant dropout, see method section). The lowly proficient bilinguals showed a mean language proficiency of 68% (*SD* = 14.7%), while the highly proficient bilinguals had a mean language proficiency of 91% (*SD* = 4.4%) on the word-recognition score. The highly proficient bilinguals thus had a similar proficiency as the German monolinguals, whose mean proficiency score was 89% (see also Table 2). The resulting proficiency groups were included in the analysis as a between-subjects factor resulting in a 3 (stimulus: *auf*, *über*, *unter*) x 2 (response direction: upward vs. downward) x 2 (proficiency: high vs. low) design. We did not distinguish between the similar- and dissimilar-OL groups in this analysis to increase power and because the groups of similar-OL and dissimilar-OL speakers did not differ significantly from each other in a preliminary analysis (interaction between stimulus, response direction, language group, and language proficiency, *F*(2, 368) = 1.37, *p* = .254, η_p_^2^ = .007). For the mean reaction times and mean error rates of the two proficiency groups see Figs 6 and 7.

#### Reaction times

The main analysis revealed no significant interaction between proficiency, stimulus, and response direction, *F*(2, 372) = 2.39, *p* = .093, η_p_^2^ = .013, but a significant interaction between stimulus and response direction, *F*(2, 372) = 17.67, *p* < .001, η_p_^2^ = .087, a significant main effect of response direction, *F*(1, 186) = 48.44, *p* < .001, η_p_^2^ = .207, as well as a significant interaction between proficiency and response direction, *F*(1, 186) = 4.71, *p* = .031, η_p_^2^ = .025. The interaction effect for proficiency and stimulus, *F*(2, 372) = 1.87, *p* = .156, η_p_^2^ = .010, as well as the main effect of stimulus, *F*(2, 372) = 1.87, *p* = .156, η_p_^2^ = .010, and the main effect of proficiency, *F* < 1, were not significant.

When we looked at the high-proficiency and the low-proficiency groups separately, we found an interaction effect for stimulus and response direction in both groups (high-proficiency group: *F*(2, 182) = 11.84, *p* < .001, η_p_^2^ = .115; low-proficiency group: *F*(2, 190) = 8.03, *p* < .001, η_p_^2^ = .078). The main effect of response direction was also significant in both groups (high-proficiency group: *F*(1, 91) = 11.67, *p* < .001, η_p_^2^ = .114; low-proficiency group: *F*(1, 95) = 41.10, *p* < .001, η_p_^2^ = .302), whereas the main effect of stimulus was not (high-proficiency group: *F*(2, 182) = 1.21, *p* = .299, η_p_^2^ = .013; low-proficiency group: *F*(2, 190) = 2.44, *p* = .090, η_p_^2^ = .025).

However, whereas in the low-proficiency group significant differences between compatible and incompatible responses were measured for all prepositions, in the high-proficiency group the differences between compatible and incompatible responses were only significant for the prepositions *auf*, and *über*, but not for *unter* (see Table 5). Interestingly, as can be seen in Fig 6, the direction of the difference between compatible and incompatible responses to the word *unter* changed. For the low-proficiency group compatible responses were slower than incompatible responses, whereas for the high-proficiency group this difference disappeared. We also conducted the same type of analysis with the variable language contact (i.e., the number of person groups with whom the children reported to communicate in German, as a group separator). We obtained very similar results as for the language-proficiency analysis, which is not surprising, as the two factors are indeed significantly correlated *r*_s_ = .28, *p* < .001. Thus, children who speak German with more person groups are also more proficient in German.

**Table 5:** Single comparisons of the RTs for compatible and incompatible trials per proficiency group.

	df	*t*	*p*
High-Proficiency-Group			
*auf*	91	-4.22	< .001
*über*	91	-3.97	< .001
*unter*	91	1.21	.230
Low-Proficiency-Group			
*auf*	95	-7.19	< .001
*über*	95	-3.79	< .001
*unter*	95	-2.57	.012

#### Error rates

Just as in the analysis of the reaction times, the interaction between proficiency, stimulus, and response direction was not significant in the error rate analysis, *F* < 1. We obtained significant effects only for the interaction between stimulus and response direction, *F*(2, 372) = 12.63, *p* < .001, η_p_^2^ = .064, the interaction between proficiency and response direction, *F*(1, 186) = 5.30, *p* = .022, η_p_^2^ = .028, and the main effect of response direction, *F*(1,186) = 10.59, *p* = .001, η_p_^2^ = .054. The interaction between proficiency and stimulus, *F*(2, 372) = 1.15, *p* = .317, η_p_^2^ = .006, the main effect of proficiency, *F*(1, 186) = 3.34, *p* = .069, η_p_^2^ = .018, and the main effect of stimulus, *F* < 1, were not significant.

When we looked at the high-proficiency and the low-proficiency group separately, we found a similar pattern as in the reaction time analysis: The interaction effect of stimulus and response direction was significant in both groups (high-proficiency group: *F*(2, 182) = 5.99, *p* = .003, η_p_^2^ = .062; low-proficiency group: *F*(2, 190) = 6.85, *p* = .001, η_p_^2^ = .067). While the main effect of response direction was significant for the low- but not for the high-proficiency group (high-proficiency group: *F* < 1; low-proficiency group: *F*(1, 95) = 12.23, *p* = .001, η_p_^2^ = .114), the main effect of stimulus was not significant in either of the groups (high-proficiency group: *F* < 1; low-proficiency group: *F* < 1).

While we found significant differences between compatible and incompatible responses for the words *auf* and *über*, but not for *unter* in the low-proficiency group, in the high-proficiency group the differences between compatible and incompatible responses were significant for the words *über*, and *unter*, but not for *auf* (see Table 6).

**Table 6:** Single comparisons of the error rates for compatible and incompatible trials per proficiency group.

	df	*t*	*p*
High-Proficiency-Group			
*auf*	91	-0.99	.326
*über*	91	-2.78	.007
*unter*	91	2.08	.040
Low-Proficiency-Group			
*auf*	95	-3.11	.002
*über*	95	-4.05	< .001
*unter*	95	0.65	.519

## Discussion

Previous studies investigating embodiment effects in L2 processing found support for the assumption that the L2 is embodied in a similar way as the L1 [35]. Furthermore, compatibility effects between word meaning and response direction suggest that experiential traces not only get reactivated during L1 word processing, but also during L2 word processing [36]. In an earlier study conducted in our lab with adult participants who acquired German either as L1 or as L2 [48], we found processing differences between the German prepositions *auf*, *über*, and *unter* depending on the native language of the speaker. L2 speakers of German whose native language uses a similar split of the upper subspace according to the feature +/- contact as German does (e.g., Russian and English) showed compatibility effects for *auf* and *über*. L2 speakers of German whose native language does not split up the upper subspace (e.g., Turkish and Korean) only showed compatibility effects for *auf*. These results suggested that the native language influences L2 processing, which is generally in line with the thinking-for-speaking framework [50].

The present study aimed at further investigating the processing of these German spatial terms in early bilingual individuals who learned German as well as at least one other language before the age of six. Just as in the previous study with adults, we compared three groups: a German monolingual group, a German bilingual group with similar OL, and a German bilingual group with dissimilar OL. We also included a wider range of other languages in the current study (Russian, English, French, Greek, etc. in the similar-OL group and Turkish, Urdu, Japanese, Thai, etc. in the dissimilar-OL group). We were particularly interested in whether we would find similar processing differences in the children as we found in the study with adult participants [48].

The results confirmed the hypothesis that experiential traces are being reactivated during word processing. All groups (i.e., both German monolinguals and German bilingual children) showed faster responses when the meaning of the preposition was compatible with the direction of the motor response (e.g., upward movement for *auf*) compared to incompatible trials (e.g., downward movement for *auf*). This finding shows that experiential traces are established and connected to words at the age of eleven to fifteen years and can be automatically accessed in a task that does not require active reading, just as was found for adult participants.

Interestingly, contrary to the adult speakers, the children did not differ across the language groups. Our results showed that bilingual children who learned German rather early in their life processed German prepositions in the same way as German monolingual children, even when their OL uses a different spatial categorization than German. According to the coactivation account, bilinguals always activate both languages [68–69]. Therefore, they constantly need to suppress the unused language, which is only possible if the speakers’ proficiency is high enough to control the two languages. As we found no significant differences between the groups, and the proficiency of the bilinguals was quite high, we can conclude that the children were indeed able to suppress their OL sufficiently to not affect the processing of the German prepositions. In addition, when we looked at the different proficiency levels directly, we found some subtle differences between highly and lowly proficient speakers. However, as the proportion of children showing a childlike L2 acquisition of German was less than 10%, we were not able to compare them directly to the early bilinguals (i.e., the simultaneous and early-successive bilinguals). In addition, language proficiency (as indicated by the word-recognition scores on the C-Tests and on our preposition test) was quite high overall, which may have lead to smaller differences between the two proficiency groups. For instance, the low-proficiency bilinguals still showed a mean proficiency score of around 70%. Nevertheless, this group was also more heterogeneous with respect to language proficiency than the high-proficiency group. For future studies, it would be interesting to look at these factors in more detail by systematically comparing performance in a sample of children with childlike L2 acquisition vs. simultaneous or early-successive bilinguals with low vs. high proficiency.

Another point worth mentioning is the fact that the effects for German native speakers were quite different for the children compared to the adults of our previous study [48]. Contrary to the adults, who showed a compatibility effect for *über*, but not for *auf*, the children showed a compatibility effect for *auf*, but not for *über*. We consider it unlikely that the reversal of this effect can be attributed to differences in the experimental procedure since both studies were very similar in this respect. Although the task was slightly altered to simplify task execution for the children (they were using only one hand and three response buttons instead of two hands and four response buttons as in the previous study), there is no obvious reason why this should lead to differential effects on *auf* and *über*. A possible explanation for the finding that children show an effect for *auf* but not for *über*, is that the word *auf* is much more frequent in the early learner input [45]. Additionally, *auf* belongs to the topological subspaces (independent of perspective) and is acquired earlier than *über*, which belongs to the coordinate-related subspaces (dependent on perspective) [70–71]. For these reasons, at the beginning of word learning the spatial experiential traces connected with *auf* might be stronger than those connected with *über*. However, it is surprising that we still found this effect in eleven to fifteen year olds, although these children had already more than ten years of German language experience.

At some point in life there seems to be a restructuring process that leads to the reversal of the effect for adults. Most likely, this will be a gradual process. For example, Thelen [72] assumes that an embodied system goes through phases of relative stability alternating with phases of relative instability. During the less stable phases, the system develops and can adapt to new circumstances. Therefore, it appears possible that the information connected in an experiential trace can also be weakened or restructured. For instance, when it comes to learning prepositions in German, generally *auf* is learned faster than *über*, due to its high frequency in the learner’s input [45] and in total use [49]. While both *auf* and *über* can be used in non-spatial configurations (e.g., *aufmachen–to open*, *übersetzen–to translate*), this is done more frequently for *auf* than for *über* [45]. Therefore, it seems possible that the connection of the spatial meaning of *auf* with the word itself is weakened throughout the lifetime as people gain more non-spatial experiences with this word. This might account for the observed differences between children and adult German speakers. When and under what circumstances the connections of experiential traces change is a question for future research. Furthermore, it should be tested whether the experiential traces for *auf* are indeed weakened as participants experience this word more frequently in non-spatial configurations. For example, this could be done in an fMRI study comparing activation for *auf* and *über* between children and adults.

An additional factor potentially influencing the results is the possible bilingualism of the German native speakers. We could not avoid including German native speakers who speak other languages in our adult study, as there are virtually no monolingual speakers in Germany. Everyone learns at least one other language in school. It therefore is possible that a later learned foreign language influences L1 processing and could contribute to the reversal of the effect in adults. We cannot rule out this explanation based on our current results, however, we can be quite sure that the majority of the foreign languages, that our German native participants learned, use the same split of the upper subspace as German does, since all participants went through the German school system, where foremost English, French and Spanish are taught as foreign languages. Future research could address the influence of particular foreign languages on the processing of prepositions in L1.

Our results also imply differences between the early acquisition of a language (children before the age of six) compared to the late acquisition of a language (adults), which is in line with research on L2 acquisition in general [52–53, 73]. For the late acquisition of a language, the L1 is probably already so strongly consolidated that it becomes difficult to learn the L2 independently of the L1 [50–51]. In addition, a truly experience-based learning situation might not be possible to obtain in an adult’s life, which might explain why the L1 is so prominent. If this explanation is correct, it might be possible to improve the learning of an L2 later in life. If a learning situation were designed to be more experiential in nature by enriching it with co-occurring multimodal experiences, it might improve the building of experiential traces connected to the L2. This in turn could facilitate the learning of the L2 in general. Additionally, it could also specifically improve the learning of spatial categorizations that differ between the L1 and the L2. For instance, research on the efficacy of gestures during the acquisition of locative prepositions [74] showed that gestures in combination with recasts enhanced the learning of locative prepositions in a delayed post-test, in contrast to the recast-only condition. The use of gestures here can be seen as one form of embodied learning, which in addition to actions are widely used to support learning in foreign language learning and especially the acquisition of foreign language words (for a review see [75]. Therefore, it would be interesting to further investigate the impact of these sorts of embodied learning strategies on L2 acquisition in future studies.

In sum, we found compatibility effects for spatial prepositions in a Stroop-like task in monolingual and bilingual children. Thus, we could confirm that the experiential traces account can be applied to language processing in children as well as to language processing in bilinguals. In addition, our study provides a good starting point to further investigate processing differences between early (before the age of six) and late (after the age of twelve) language learners as well as between highly and lowly proficient speakers of German with respect to experiential traces.

## Supporting information

S1 Table Language categorization. (PDF)

S1 File Data. (CSV)
