# Supplementary material for: How do German bilingual schoolchildren process German prepositions? – A study on language-motor interactions
Source: PLoS One. 2018 Mar 14;13(3):e0193349. doi: 10.1371/journal.pone.0193349 (PMC5851577; doi:10.1371/journal.pone.0193349)
Supplement: S1 Table — (PDF) [file pone.0193349.s001.pdf]

Language group categorization		
Similar OL	Dissimilar-OL	Not classifiable
Albanian	Bahasa	Egyptian-Arabic
Bosnian	Japanese	Libyan-Arabic
Chechen	Korean	Morrocan-Arabic
Croatian	Lingala	Eritrean
Dari	Pashtu	Ghanaian
English	Philippine	Kotokoli
Farsi	Swahili	Nigerian
French	Thai	
Greek	Turkish	
Hungarian	Urdu	
Italian		
Kurdish		
Modern Standard Arabic		
Polish		
Portuguese		
Punjabi		
Romanes		
Rumanian		
Russian		
Serbian		
Slovakian		
Syrian-Arabic		
Tunisian-Arabic		
Twi		
Vietnamese		
